# Contrasting effects of afferent and efferent vagal nerve stimulation on insulin secretion and blood glucose regulation

**DOI:** 10.14814/phy2.12718

**Published:** 2016-02-16

**Authors:** Erin E. Meyers, Ana Kronemberger, Vitor Lira, Kamal Rahmouni, Harald M. Stauss

**Affiliations:** ^1^ Department of Health and Human PhysiologyThe University of IowaIowa CityIowa; ^2^ Department of PharmacologyThe University of IowaIowa CityIowa

**Keywords:** Blood pressure, diabetes, glucagon, heart rate, rats

## Abstract

Parasympathetic activation reduces hepatic glucose release and increases pancreatic insulin secretion in hyperglycemic conditions. Thus, vagal nerve stimulation (VNS) may potentially be effective in treating type II diabetes. To investigate this possibility, we hypothesized that VNS reduces blood glucose concentration [Glu] via insulin secretion. [Glu] together with insulin and glucagon serum concentrations were determined in anesthetized rats during baseline conditions and 120 min of cervical VNS with the nerve left intact for combined afferent and efferent VNS (*n* = 9) or the nerve sectioned proximal or distal from the stimulation electrode for selective efferent (*n* = 8) or afferent (*n* = 7) VNS, respectively. Afferent VNS caused a strong and sustained increase in [Glu] (+108.9 ± 20.9% or +77.6 ± 15.4%, after 120 min of combined afferent and efferent VNS or selective afferent VNS) that was not accompanied by an increase in serum insulin concentration. However, serum insulin levels increased significantly with selective efferent VNS (+71.2 ± 27.0% after 120 min of VNS) that increased [Glu] only temporarily (+28.8 ± 11.7% at 30 min of VNS). Efferent VNS initially increased serum glucagon concentration which remained elevated for 120 min when efferent VNS was combined with afferent VNS, but returned to baseline with selective efferent VNS. These findings demonstrate that afferent VNS causes a marked and sustained increase in [Glu] that is partly mediated by suppression of pancreatic insulin secretion. In contrast, efferent VNS stimulates pancreatic glucagon secretion that appears to be antagonized by insulin secretion in the case of selective efferent VNS. Selective efferent VNS may potentially be effective in treating type II diabetes.

## Introduction

More than half a century after the first implantation of a cardiac pacemaker in 1958 (Aquilina 2006) there is a renewed interest in bioelectronics and specifically in devices that stimulate peripheral activity to relieve clinical conditions (Anonymous, 2014). Implantable vagal nerve stimulators belong to this class of devices, and the first human vagal nerve stimulator was implanted in 1988 for the treatment of seizures (Penry and Dean 1990). Since then, chronic cervical vagal nerve stimulation (VNS) has emerged as a new tool to treat a variety of other human diseases besides epilepsy, including major depression (Rush et al. 2000) and obesity (Ogbonnaya and Kaliaperumal 2013; Bodenlos et al. 2014; Chiu and Soffer 2015). Specifically, substantial weight loss has been observed in patients treated with chronic VNS for therapy‐refractory epilepsy (Burneo et al. 2002) or major depression (Bodenlos et al. 2007; Pardo et al. 2007). Because obesity is one of the most important predisposing conditions leading to type II diabetes chronic cervical VNS may be beneficial in patients with impaired glucose tolerance, metabolic syndrome, or overt type II diabetes. This possibility is further supported by the metabolic effects of the parasympathetic nervous system. Ribeiro et al. (Ribeiro et al. 2015) demonstrated that insulin acting on the central nervous system causes a reduction in hepatic glucose release that is accompanied by an increase in subdiaphragmatic vagal nerve activity in normotensive rats. Similarly, Tanida et al. (Tanida et al. 2015) found a marked reduction in blood glucose concentration during direct electrical stimulation of hepatic parasympathetic nerve fibers in mice. Besides inhibition of hepatic glucose release, the vagus nerve also controls secretion of insulin and glucagon from the islets of Langerhans. Electrical stimulation of the distal end of the sectioned subcardiac thoracic vagal nerve increased pancreatic release of insulin and glucagon in pigs (Holst et al. 1981a) and dogs (Ahren and Taborsky 1986). This response is largely dependent on the prevailing blood glucose concentration, because high glucose levels caused greater insulin release while low blood glucose concentrations caused greater glucagon release in response to efferent VNS (Holst et al. 1981a). Collectively, these studies demonstrate that efferent vagal nerve activity directed to the liver inhibits hepatic glucose release and efferent vagal activity directed to the islets of Langerhans not only stimulates insulin secretion in hyperglycemic conditions but can also stimulate glucagon secretion in hypoglycemia.

While the hypoglycemic effects of efferent parasympathetic nervous system activity are reasonably well established, the effects of afferent parasympathetic pathways conveying sensory information from splanchnic organs including the liver and the pancreas to the central nervous system on glucose metabolism are less understood. Retrograde tracing techniques have clearly identified afferent parasympathetic innervation of the liver (Magni and Carobi 1983) and the islet cells (Neuhuber 1989). Direct nerve recordings from afferent fibers from hepatic branches of the vagus nerve revealed an inverse relationship between the glucose concentration in the portal vein and afferent hepatic vagal nerve activity (Niijima 1984), which may signal low glucose levels to the brain and initiate food intake (Jensen et al. 2013). Iwasaki et al. (Iwasaki et al. 2013) found that vagal afferent neurons innervating the pancreas are sensitive to insulin and may convey changes in pancreatic and possibly systemic insulin levels to the brain. While much can be learned from these and similar studies, the net effect of vagal afferent signaling to the central nervous system on glucose metabolism still remains unknown.

The best established clinical application of VNS is for the treatment of therapy‐refractory epilepsy (FDA approval in 1997) and major depression (FDA approval in 2005). For these applications, the cervical vagus nerve is electrically stimulated. While the mechanisms underlying the therapeutic effects of VNS in these conditions are not well understood, they are thought to be initiated through activation of afferent fibers projecting to the nucleus of the solitary tract (Ogbonnaya and Kaliaperumal 2013). Nevertheless, cervical VNS is likely to activate both afferent and efferent pathways as indicated by side effects that can be explained by efferent VNS, such as bronchoconstriction (Bijwadia et al. 2005; Gschliesser et al. 2009). Even though cervical VNS has been employed in patients for over 20 years, to the best of our knowledge, its potential effects on glucose metabolism have never been evaluated in humans. While efferent cervical VNS may elicit a hypoglycemic effect through inhibition of hepatic glucose release and stimulation of pancreatic insulin secretion, afferent cervical VNS may falsely signal low glucose levels in the portal vein and high insulin concentrations in the pancreas to the brain, which may initiate hyperglycemic responses, such as hepatic glucose release and inhibition of insulin secretion.

To investigate these potentially contrasting effects of efferent versus afferent cervical VNS on glucose metabolism, we studied the effects of selective efferent and afferent cervical VNS on glucose metabolism in an acute study in anesthetized rats. Our hypothesis was that efferent cervical VNS decreases blood glucose concentration, while afferent cervical VNS increases blood glucose concentration. As an experimental approach, we recorded blood glucose concentration, and serum insulin and glucagon levels before and during 120 min of continuous cervical VNS with the vagus nerve intact or sectioned proximal or distal from the stimulation electrode for selective efferent or afferent VNS, respectively. Importantly, our results demonstrate differential effects of afferent versus efferent cervical VNS on insulin secretion and glycemia.

## Methods

### Animals

Experiments were performed in male normotensive Sprague Dawley rats at an age of 120 ± 4 days (395 ± 9 g body wt). Rats were housed in clear plastic cages, and temperature and light periods (12‐h light–dark cycle; light on between 6:00 am and 6:00 pm) were controlled. A standard rat chow and tap water were provided ad libitum. Experiments were approved by the Institutional Animal Care and Use Review Committee of the University of Iowa.

### Instrumentation

Initially, rats were anesthetized using isoflurane in room air. A telemetric glucose sensor (HD‐XG, DSI, St. Paul, MN) was inserted in the abdominal aorta via the right femoral artery for continuous blood glucose concentration monitoring. Then, through a midline neck incision, a catheter was inserted in the left common carotid artery for recording of arterial blood pressure and heart rate. Bipolar stimulation electrodes were placed around the right cervical vagus nerve and electrically insulated from surrounding tissue using silicon elastomer (Kwik‐Sil, World Precision Instrument, Inc., Sarasota, FL).

### Experimental protocol

After instrumentation, anesthesia was maintained at 1.2–1.5% isoflurane in oxygen. Blood pressure and heart rate were recorded by connecting the arterial catheter to a pressure transducer (P23 ID, Gould‐Statham, Oxnard, CA) and amplifier (Series 4000, Gould, Inc., Cleveland, OH). The output of the amplifier was connected to an A/D‐converter (ADUSB4CH, Harald Stauss Scientific, Iowa City, IA) for data acquisition using the WinAD module of the freely available HemoLab software (http://www.haraldstauss.com/HaraldStaussScientific/hemolab). Blood glucose concentration was recorded using the Dataquest A.R.T. software (Version 4.35, DSI, Saint Paul, MN).

**Figure 1:**
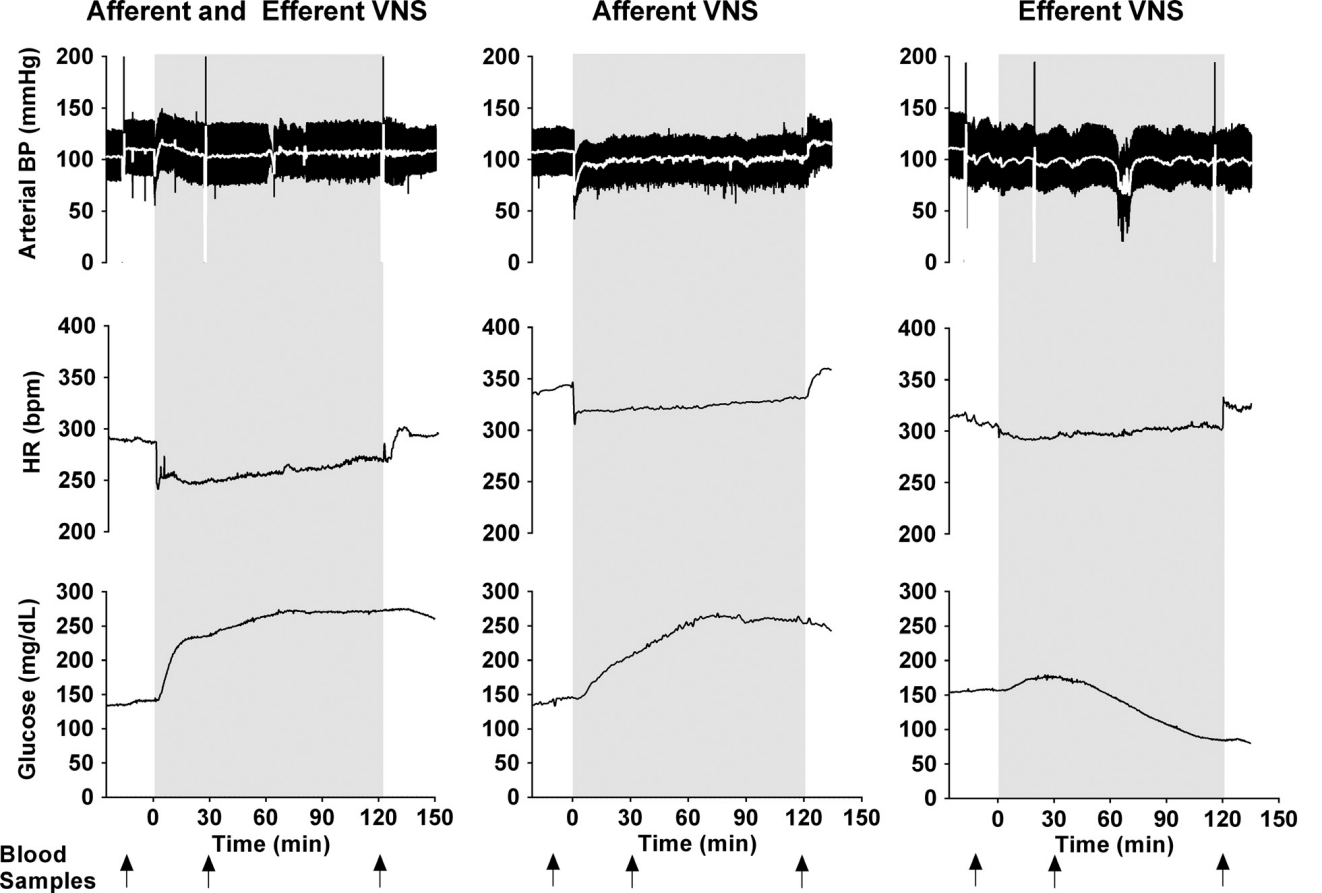
Typical responses to combined afferent and efferent vagal nerve stimulation (VNS, left) and to selective afferent (middle) or efferent (right) VNS. From top to bottom recordings of arterial blood pressure (BP), heart rate (HR), and blood glucose concentration are shown. Blood samples for glucose, insulin, and glucagon measurements were taken at baseline before initiation of VNS and at 30 min and 120 min of VNS (arrows).

The experimental protocol consisted of three experimental conditions as illustrated in Figure 1. In nine animals, the vagal nerve was left intact for combined afferent and efferent stimulation. In eight animals, the nerve was sectioned proximal from the stimulation electrode for selective efferent vagal nerve stimulation and in seven animals the vagal nerve was sectioned distal from the stimulation electrode for selective afferent nerve stimulation. After establishing stable baseline conditions, continuous VNS (rectangular impulses at 5 Hz, 3 V, 1 msec pulse width, Stimulator SD9, Grass Instruments, Warwick, RI) was initiated and maintained for 2 h. The stimulator was then turned off, and the animals were euthanized. Blood samples were obtained from the arterial line for the measurement of blood glucose concentration as well as insulin and glucagon serum concentrations at baseline before VNS and at 30 min and 120 min after the initiation of VNS.

### VNS parameters

We used rectangular impulses of 1 msec pulse width and 3 V voltage, delivered at a frequency of 5 Hz. These stimulation parameters were selected based on pilot experiments that indicated that these stimulation parameters represent the lowest stimulation intensity (combination of voltage, pulse width, and frequency) that resulted in an immediate and consistent bradycardic response. The bradycardic response was used to verify the biological effectiveness of the stimulation and can be seen in Figure 1. The vagus nerve contains A, B, and C fibers according to the classification of Gasser and Erlanger (Gasser and Erlanger 1930). On the basis of an estimated electrode resistance between 2000 Ω and 4000 Ω, we estimate that the stimulation current was in the range of 0.75–1.5 mA, which would be high enough to activate at least some C fibers and the larger A and B fibers that have a smaller activation threshold than the smaller C fibers (Groves and Brown 2005). Clinically, stimulation parameters of 20–30 Hz frequency, 0.25–0.5 msec pulse width, and 1.0–1.5 mA (up to a maximum of 3.5 mA) current are typically used (Labiner and Ahern 2007). Thus, a lower stimulation frequency but a longer pulse width with similar stimulation current was used in our study than what is typically used in clinical applications.

### Biochemical analyses

Blood glucose concentrations were determined using a TRUEtrack glucose meter (Nipro Diagnostics, Fort Lauderdale, FL). The implanted telemetric blood glucose sensor provides an electrical current proportional to glucose concentration. The glucose values obtained from the TRUEtrack glucose meter were used to calibrate this electrical current into glucose concentration values. Serum insulin and glucagon concentrations were quantified using commercially available ELISA kits (Kit #90010 for insulin and Kit #81505 for glucagon, CrystalChem, Downers Gove, IL).

### Data analyses

Blood glucose data collected using the Dataquest A.R.T. software were combined and synchronized in time with the blood pressure and heart rate data collected using the WinAD software and then analyzed together using the Analyzer module of the HemoLab software. For each animal, values for mean arterial blood pressure, heart rate, and blood glucose concentration were extracted at baseline (before VNS), and at 30 min and 120 min of VNS.

### Statistics

Data provided in the text of the manuscript are presented as arithmetic mean values ± SEM. Data presented in figures are shown as box‐and‐whisker plots with the median, quartiles, and extreme values. Separate statistical analyses were performed for each experimental condition (combined afferent and efferent VNS, selective afferent VNS, and selective efferent VNS). Statistical comparison between the three time points (baseline before VNS and 30 min and 120 min of VNS) were done by one‐way analysis of variance for repeated measures with post hoc Fisher's tests. Statistical significance was assumed for *P* ˂ 0.05.

## Results

Figure 1 shows typical examples of the blood pressure, heart rate, and blood glucose concentration responses to VNS in the three experimental conditions (combined afferent and efferent VNS and selective afferent or efferent VNS). Afferent VNS (either as combined afferent and efferent VNS or as selective afferent VNS) caused a strong and sustained increase in blood glucose concentration. In contrast, with selective efferent VNS blood glucose concentration increased only temporarily and returned to baseline or even decreased by more than 10 mg/dL below baseline in five of the eight animals after 120 min of VNS.

### Hemodynamic responses to VNS

**Figure 2:**
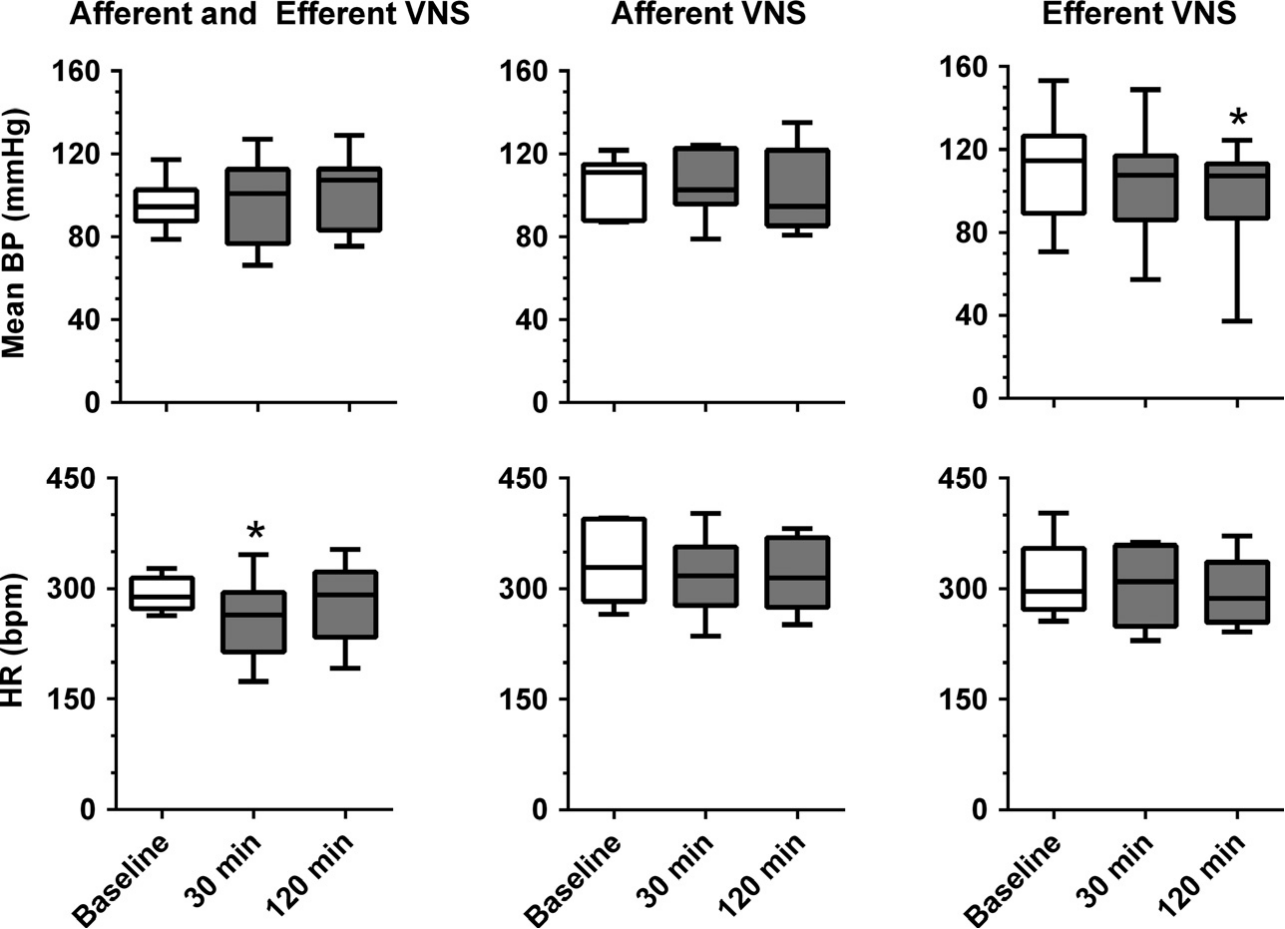
Mean arterial blood pressure (BP) and heart rate (HR) before vagal nerve stimulation (VNS, Baseline) and at 30 min and 120 min after initiation of combined afferent and efferent VNS (left, n = 9) and selective afferent (middle, n = 7) or efferent (right, n = 8) VNS. *P < 0.05 versus Baseline.

Arterial blood pressure and heart rate were recorded to verify the responsiveness of VNS through an immediate bradycardic response (as shown in Fig. 1) and to ensure stable cardiovascular conditions during anesthesia. At the 30 min and 120 min time points of VNS no significant changes from baseline were observed for mean blood pressure in either the combined afferent and efferent VNS or selective afferent VNS conditions. With selective efferent VNS, a small decrease in blood pressure was observed after 120 min of VNS (Fig. 2, top). In all animals under the three experimental conditions, heart rate initially decreased when the stimulator was turned on but gradually returned to baseline levels. The initial decrease in heart rate was accompanied by a brief and transient hypotensive response (Fig. 1). In the selective afferent and efferent VNS conditions, heart rate had returned to baseline already after 30 min of stimulation, whereas in the combined afferent and efferent VNS condition, heart rate had only returned to its baseline level by 120 min of VNS (Fig. 2, bottom).

### Blood glucose regulation during VNS

**Figure 3:**
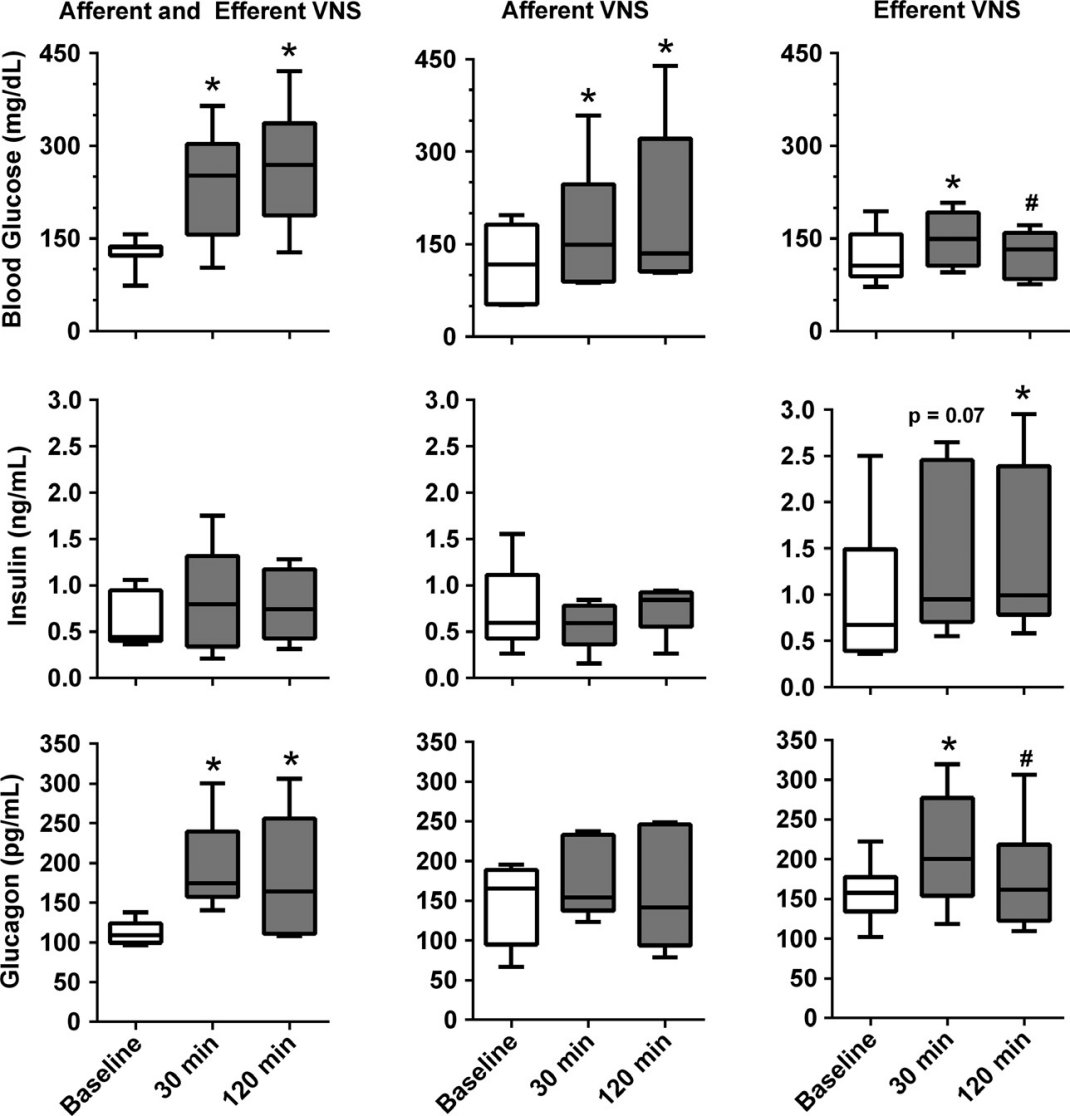
Blood glucose concentration (top) and serum insulin (middle) and glucagon (bottom) concentrations before vagal nerve stimulation (VNS, Baseline) and at 30 min and 120 min after initiation of combined afferent and efferent VNS (left, n = 9 for glucose, n = 5 for insulin and glucagon) and selective afferent (middle, n = 7 for glucose, n = 5 for insulin and glucagon) or efferent (right, n = 8 for glucose, n = 6 for insulin and glucagon) VNS. *P < 0.05 versus Baseline. ^#^P < 0.05 versus 30 min.

Selective afferent VNS or combined afferent and efferent VNS caused a strong and sustained increase in blood glucose concentration. Compared to baseline, after 30 min of VNS blood glucose concentration increased to 182.7 ± 18.9% (combined afferent and efferent VNS) and 146.8 ± 10.0% (selective afferent VNS), increasing further at 120 min of VNS to 208.9 ± 20.9% and 177.6 ± 15.4%, respectively (Fig. 3). In contrast, selective efferent VNS increased blood glucose concentration only temporarily (+28.8 ± 11.7% at 30 min) followed by a return to baseline levels (Fig. 3).

Despite the substantial increase in blood glucose concentration, insulin serum levels did not increase significantly with either combined afferent and efferent VNS or selective afferent VNS, suggesting that afferent VNS suppresses insulin secretion (Fig. 3). In contrast, with selective efferent VNS, insulin levels increased throughout the 120 min of VNS (+57.1 ± 17.4% at 30 min and +71.2 ± 27.0% at 120 min) and this increase in insulin serum levels reached statistical significance at 120 min of VNS.

Serum glucagon concentration significantly increased by 30 min of efferent VNS (+72.1 ± 14.5% and +31.6 ± 8.3% for combined afferent and efferent VNS and selective efferent VNS, respectively). Serum glucagon concentration remained elevated for the remainder of the 120 min of VNS with combined afferent and efferent VNS (+57.6 ± 23.4%), but returned to baseline with selective efferent VNS (Fig. 3). In contrast to efferent VNS, selective afferent VNS did not increase serum glucagon concentration (Fig. 3).

## Discussion

Previous studies on parasympathetic control of glucose metabolism have largely focused on the efferent parasympathetic innervation of the liver and pancreas, while the role of afferent parasympathetic pathways has received considerably less attention. Early on, these experimental studies utilized electrical stimulation of the distal end of sectioned vagal nerve fibers anatomically located close to these effector organs (Shimazu 1967; Shimazu and Amakawa 1968; Holst et al. 1981a; Ahren and Taborsky 1986). Clinically, however, the most frequently used approach for VNS consists of electrical stimulation of the intact cervical vagus nerve. Thus, our study was designed to investigate a possible differential effect of afferent versus efferent cervical VNS on glucose metabolism. The most important and potentially novel finding of our study is that afferent cervical VNS caused a strong and sustained increase in blood glucose concentration that was not accompanied by an increase in serum insulin concentration, suggesting that inhibition of insulin secretion has at least partly contributed to the strong and sustained hyperglycemic effect of afferent cervical VNS.

Combined afferent and efferent VNS caused severe and sustained hyperglycemia without stimulating insulin secretion. This sustained hyperglycemia can be attributed to afferent VNS because a similarly sustained glucose response was observed with selective afferent VNS but not with selective efferent VNS. Surprisingly, the marked and sustained hyperglycemia with VNS (combined afferent and efferent or selective afferent) failed to trigger an increase in serum insulin concentration. Thus, in contrast to efferent VNS that has been demonstrated by us in this study and by others (Holst et al. 1981a; Ahren and Taborsky 1986) to stimulate insulin secretion, afferent VNS inhibits insulin secretion from the endocrine pancreas. A potential negative feedback loop by which afferent VNS may inhibit insulin secretion may start and end at the endocrine pancreas. It has been demonstrated that nodose ganglion neurons of vagal afferents originating from the endocrine pancreas are activated by insulin and may serve to convey changes in pancreatic insulin levels to the brain (Iwasaki et al. 2013). These insulin‐responsive vagal afferent fibers activate neurons in the nodose ganglion that supposedly project to the nucleus of the solitary tract (NTS) from where projections originate to hypothalamic areas, such as the arcuate and paraventricular nuclei (PVN), which are involved in regulation of food intake and autonomic nervous system activity (Morton et al. 2006). It is possible that these pathways activate pre‐sympathetic neurons (e.g., in the PVN) projecting to the rostral ventrolateral medulla (RVLM) and intermediolateral cell column of the spinal cord (IML) (Saper et al. 1976; Swanson and Kuypers 1980; Luiten et al. 1985; Badoer 1996; Morton et al. 2006) and through the sympathetic splanchnic nerve back to the endocrine pancreas where sympathetic splanchnic nerve fibers have been demonstrated to inhibit insulin secretion (Holst et al. 1981b; Andersson et al. 1982). In short, our results indicate that afferent VNS potentially mimics the physiologic sensory afferent parasympathetic activation originating in the pancreas when insulin secretion is elevated, thereby resulting in the subsequent inhibition of insulin secretion via a compensatory sympathetic stimulation of the endocrine pancreas. However, afferent parasympathetic pathways originating from the liver may have also contributed to the inhibitory effect of afferent VNS on insulin secretion. This is supported by the demonstration by Lee and Miller (Lee and Miller 1985) that electrical stimulation of the central end of the sectioned hepatic vagus nerve suppressed insulin secretion.

The suppression of insulin secretion by afferent VNS is consistent with the sustained hyperglycemia observed with combined afferent and efferent VNS and with selective afferent VNS. However, other mechanisms have likely contributed to the initial increase in blood glucose concentration observed in all three experimental conditions (combined afferent and efferent VNS and selective afferent or efferent VNS). In the case of efferent VNS (either selective or in combination with afferent VNS) the initial increase in blood glucose concentration may be the direct result of parasympathetic innervation of pancreatic *α* cells based on the findings that VNS triggers glucagon release (Bloom and Edwards 1981; Holst et al. 1981a). Consistent with these studies, our results demonstrate a marked and significant increase in serum glucagon concentration with efferent VNS. This increase in serum glucagon concentration was sustained and lasted for the whole 120 min stimulation period with combined efferent and afferent VNS but was only transient with selective efferent VNS. Since insulin acts as a strong inhibitor for glucagon release from *α* cells (Holst et al. 1981b; Kaneko et al. 1999; Kawamori et al. 2009) it is reasonable to assume that the increase in insulin serum levels with selective efferent VNS explains the transient nature of the serum glucagon time course with selective efferent VNS. Conversely, the suppression of insulin release with afferent VNS prevents the insulin‐mediated inhibition of glucagon release from *α* cells, contributing to the increased blood glucose levels observed with combined afferent and efferent VNS.

Interestingly, the increase in blood glucose levels with selective afferent VNS was not accompanied by an increase in serum glucagon concentration. Thus, other mechanisms are likely to be involved, such as hepatic glucose release triggered by the sympathetic nervous system (Shimazu 1967; Shimazu and Amakawa 1968; Järhult et al. 1980; Tanida et al. 2015) that may be activated through afferent VNS via pathways outlined above (Saper et al. 1976; Swanson and Kuypers 1980; Luiten et al. 1985; Badoer 1996; Morton et al. 2006). In this regard, Järhult et al. (Järhult et al. 1980) demonstrated in anesthetized cats that electrical stimulation of hepatic sympathetic nerves increased blood glucose concentration only modestly, while combined sympathetic stimulation to the liver and to the pancreas caused a strong and significant increase in blood glucose concentration. Importantly, this increase in blood glucose concentration with combined hepatic and pancreatic sympathetic stimulation was accompanied by a decrease in serum insulin concentration (Järhult et al. 1980). Thus, the increase in blood glucose concentration with selective afferent VNS observed in our study may likewise be the consequence of sympathetic‐mediated hepatic glucose release in combination with sympathetic‐mediated inhibition of insulin release from pancreatic *β* cells.

A limitation of this study is that it was performed in isoflurane‐anesthetized rats. Isoflurane at a concentration of 2% has been found to inhibit insulin secretion from rat pancreatic islets of Langerhans (Desborough et al. 1993). Although we kept the isoflurane concentration below 1.5% we cannot exclude the possibility that the inhibition of insulin release observed with afferent VNS may partly be related to the isoflurane anesthesia. However, efferent VNS still increased insulin serum levels significantly, demonstrating that the isoflurane anesthesia has not completely blocked insulin release. Nevertheless, it is possible that isoflurane anesthesia has reduced baseline insulin secretion, blunting any further decreases in insulin serum levels in response to afferent VNS. Thus, the possibility exists that in conscious rats, afferent stimulation may suppress insulin secretion to a point where serum levels fall below baseline levels. In a different study, in which we investigate the effects of chronic VNS on hypertension‐induced cardiovascular end‐organ damage, we obtained blood glucose readings during VNS in a conscious spontaneously hypertensive rat. Preliminary data (unpublished) indicate that combined afferent and efferent VNS increases blood glucose concentration not only in the anesthetized state, where insulin secretion may be inhibited by the anesthesia, but also in the conscious state.

Considering that cervical VNS is used as an approved treatment for therapy‐refractory epilepsy and major depression and further considering that afferent pathways are thought to mediate the therapeutic effects of VNS in these conditions (Ogbonnaya and Kaliaperumal 2013), the hyperglycemic effect of afferent cervical VNS identified in this study may potentially be clinically relevant. Our experiments in anesthetized rats may not directly translate to the situation in patients receiving VNS therapy. Nevertheless, the hyperglycemia in combination with the inhibited insulin secretion in response to afferent VNS in our study may justify investigating the effects of VNS on glucose metabolism in patients receiving VNS therapy which – to our knowledge – has not been investigated so far. On the other hand, if it were possible to design stimulating devices that selectively stimulate efferent vagal nerve fibers, VNS may potentially be an option for the treatment of insulin resistance, metabolic syndrome, or even type II diabetes.

In conclusion, the results of this study demonstrate that cervical VNS (combined afferent and efferent VNS) increases blood glucose concentration in anesthetized rats and suggests that afferent but not efferent VNS suppresses pancreatic insulin secretion. Further studies are warranted to investigate if chronic VNS (e.g., in patients with epilepsy or major depression) reduces glucose tolerance by suppression of insulin release via afferent VNS. Likewise, it appears important to further investigate the potential use of selective efferent VNS in the control of blood glucose levels in prediabetic and diabetic states.

## Conflict of Interest

None declared.
